# Geo-epidemiology of temporal artery biopsy-positive giant cell arteritis in Australia and New Zealand: is there a seasonal influence?

**DOI:** 10.1136/rmdopen-2017-000531

**Published:** 2017-08-29

**Authors:** Elisabeth De Smit, Linda Clarke, Paul G Sanfilippo, Tony R Merriman, Matthew A Brown, Catherine L Hill, Alex W Hewitt

**Affiliations:** 1 Department of Ophthalmology, Centre for Eye Research Australia, The University of Melbourne, Royal Victorian Eye and Ear Hospital, East Melbourne, Victoria, Australia; 2 Department of Biochemistry, School of Biomedical Sciences, University of Otago, Dunedin, New Zealand; 3 Institute of Health and Biomedical Innovation, Queensland University of Technology, Translational Research Institute, Princess Alexandra Hospital, Brisbane, Queensland, Australia; 4 Department of Rheumatology, The Queen Elizabeth Hospital, Adelaide, South Australia, Australia; 5 School of Medicine, Menzies Research Institute Tasmania, University of Tasmania, Hobart, Tasmania, Australia; 6 Lions Eye Institute, University of Western Australia, Perth, Western Australia, Australia

**Keywords:** giant cell arteritis, temporal arteritis, epidemiology, environment, geography, risk factor, seasons, latitude, temporal artery biopsy

## Abstract

**Objective:**

Previous studies, although inconclusive, have suggested possible associations of environmental risk factors with the development of giant cell arteritis (GCA). We aim to investigate seasonal influence on the incidence of GCA across Australia and New Zealand.

**Methods:**

In establishing an international study to investigate the molecular aetiology of GCA, archived temporal artery biopsy (TAB) specimens primarily from Australia and New Zealand were obtained. Demographic details including age, sex and date of TAB were collected from collaborating pathology departments. The season in which GCA was diagnosed was determined and compared with previous reports investigating the association between environmental risk factors and GCA.

**Results:**

Our study comprises data from 2224 TAB-positive patients with GCA; 2099 of which were from patients in Australia and New Zealand. The mean age at time of diagnosis was 76.4 years of age. The female-to-male ratio was 2.2:1. We noted equal distribution of the incidence rate across all four seasons (530–580 cases diagnosed every quarter). Statistical analysis of seasonal variation by Poisson regression and cosinor methods showed no incidence preponderance across seasons. Our results do not support a seasonal component contributing to the onset of disease. Our literature search identifies no consistent environmental risk factor in association with GCA.

**Conclusion:**

This is the largest GCA data set reported outside of Europe. Our results demonstrate equal distribution of the incidence rate across all four seasons. In contrast to some earlier reports, we did not identify evidence of a seasonal component contributing to the onset of disease.

**Key messages**

**What is already known about this subject?**

- Environmental factors have been implicated in the development of giant cell arteritis (GCA). Yet previous study findings have been inconsistent and results inconclusive.

**What does this study add?**

- With this geoepidemiological study, we sought to identify whether there was indeed a seasonal trend in the onset of GCA.
- We reviewed the demographic data of 2224 obtained temporal artery biopsy samples, the largest data set in Australasia. We found no statistically significant difference between seasons with regard to the onset of disease.
- The results of our systematic literature review searching for published associations between environmental risk factors and GCA highlight that there is no convincing and certainly no consistent evidence for the role of direct environmental factors on the risk of developing GCA.

**How might this impact on clinical practice?**

- Our data suggest that seasonality should not be viewed as a risk factor for GCA in clinical practice. As such the month in which a patient presents should not influence a clinician’s decision as to whether the patient is likely to have GCA.

## Introduction

Giant cell arteritis (GCA) is the most common chronic systemic inflammatory vasculitis affecting people aged over 50 years.^1^ This granulomatous vasculitis primarily targets the vessels in the head and neck. The ophthalmic artery is commonly affected leading to visual loss and as such it is one of the few true ophthalmic emergencies. The gold standard method to diagnose GCA is through temporal artery biopsy (TAB).^2^

The incidence of GCA rises markedly with increasing age, peaking in the seventh decade of life.^3 4^ GCA is most prevalent in populations of Scandinavian ancestry. However, GCA has been reported in people of African, Asian, Hispanic and Arab descents, although incidence among these populations has not been clearly studied.^2^ Given that GCA primarily affects people of Northern European ancestry, there is clear evidence for a role of genetic factors in GCA.^5–7^

Geographical variation, seasonal fluctuations and cyclic patterns have been observed in the incidence of GCA.^1^ As a result, theories have emerged suggesting an environmental, including possibly an infectious aetiology.^8–13^ The relative contribution of genetic and environmental factors as an explanation for geographical differences in GCA incidence remains disputed.^14 15^

In establishing an international study to investigate the molecular aetiology of GCA, archived TAB specimens were obtained. Data were collated and analysed from pathology centres primarily around Australia and New Zealand. Herein we sought to investigate the potential pathogenic influence of seasonality on GCA. In addition, we report a systematic review of the literature exploring the potential non-infectious environmental risk factors associated with GCA.

## Methods

A total of 28 pathology centres from across Australia and New Zealand were approached to participate in this study.^16^ In addition, two sites in Europe collaborated on this project. Ethics approval was obtained for all participating sites (online supplementary appendix A).^16^ Each centre facilitated the collation of details regarding their archived TAB formalin-fixed paraffin-embedded (FFPE) specimens.

10.1136/rmdopen-2017-000531.supp1 Supplementary file 1

Only TAB samples demonstrating histological evidence of GCA as defined by ‘inflammation (granulomatous or mixed inflammatory cell infiltrate) of the arterial wall with fragmentation and disruption of the internal elastic lamina’ were included. Inflammation could also involve the media, adventitia and perivascular space (vasa vasorum). Multinucleated giant cells may or may not be present. Histology reports were obtained and reviewed for inclusion criteria. As such we could also ensure that the histology recruitment criteria conducted at each centre were uniform. Demographic data as well as specimen details were collected on all cases.

Pathologists were each sent a study sample collection proforma to complete. Data collected on these forms included all relevant details per specimen retrieved: the patient’s gender, their date of birth (DOB), the date of TAB (DOT) procedure and the hospital location at which this procedure was conducted. Pathologists screened their local databases to select appropriate cases and provided us with the basic data. Given the large volume of cases, the patients’ medical records were not requested or assessed for detailed clinical information.

The age of patient at time of diagnosis was calculated based on the difference between DOB and DOT. Using DOT available, we categorised patients by the representative season in which they were diagnosed. For all states within Australia as well as for all participating sites in New Zealand, ‘spring’ meant that a patient had undergone a TAB procedure during September–November, ‘summer’ during December–February, ‘autumn’ during March–May and ‘winter’ during June–August. As we had insufficient data from the Northern Territory, Northern Queensland or northern parts of Western Australia, we did not use wet or dry season classifications. For European countries, the reverse months and seasons were used.

We investigated the relationship between GCA rate and seasonal variation by fitting two types of generalised linear model. In the first instance, we modelled the number of GCA events as a Poisson response with a log link function. Season was included as the main effect with age and gender as categorical covariates. Seasonal trends in GCA rate were also evaluated by performing cosinor analysis using the ‘R’ software plug-in ‘season.’ The cosinor model assumes a sinusoidal pattern with the amplitude describing the size of the seasonal change and the phase, its peak. The sinusoid assumes a smooth seasonal pattern that is symmetric about its peak (so the rate of the seasonal increase in disease is equal to the decrease). Age and sex were included as covariates. In both types of analyses, models were evaluated for goodness of fit.

In addition to our study results, a systematic review of publications up to February 2017 was performed using the PubMed and ISI Web of Science databases. The search criteria included: ‘Giant Cell Arteritis OR temporal arteritis’ AND ‘Country OR geoepidemiology OR seasons OR socioeconomic OR environment OR climate OR weather OR cyclic OR sunlight OR latitude OR altitude’. Articles written in English, French or Dutch were reviewed for relevance. PRISMA (Preferred Reporting Items for Systematic Reviews and Meta-Analyses) guidelines were followed.^17^

Selected studies fulfilled the following criteria: a diagnosis of GCA was defined as either having positive TAB, a clearly established clinical definition, or meeting the American College of Rheumatology classification clinical criteria for GCA, and that the study of interest explored the association of a specific environmental trigger in the context of GCA disease. Articles were excluded if they did not distinguish between GCA and polymyalgia rheumatic, if they were reviews or if they employed data from other studies to explore a larger cohort. When studies were updates of previous cohorts, the most recent study was used and the most current data were reviewed.

## Results

### Study results

Data from 2224 patients with histologically confirmed GCA were included; 1881 samples belonged to patients from Australia, 218 from New Zealand and 125 from Germany and the Netherlands. The breakdown of the sample sizes by states and territories within Australia and New Zealand is displayed in table 1. The majority of the pathology centres participating in this study were based in the Southern States in Australia and as such more cases were recruited from Victoria, South Australia and New South Wales.

**Table 1:** Location, latitude and number of giant cell arteritis cases recruited

Location	Latitude	Number of cases
Australia		1881
Victoria (Melbourne)	−37.813628	731
New South Wales (Sydney)	−33.868820	467
Queensland (Brisbane)	−27.469771	79
Tasmania (Hobart)	−42.882138	83
South Australia (Adelaide)	−34.928499	290
Western Australia (Perth)	−31.950527	199
Australian Capital Territory (Canberra)	−35.280937	32
New Zealand		218
Wellington (Wellington)	−41.286460	57
Canterbury (Christchurch)	−43.532054	92
Otago (Dunedin)	−45.878760	69
Europe		125
The Netherlands (Rotterdam)	51.924420	98
Germany (Heidelberg)	49.398752	27
Total		2224

Latitude coordinates from http://www.latlong.net.

The TAB FFPE specimens recruited for our GCA Genome Wide Association Study (GWAS) dated between 1987 and 2015 with over 80% of samples belonging to patients having undergone their biopsy procedure between 2000 and 2015 (online supplementary figure 1). This is likely to represent an artefact reflecting the searchability of hospital databases rather than any true increase in incidence in the last 10–15 years.

10.1136/rmdopen-2017-000531.supp2 Supplementary file 2

Participants included 1538 female and 686 male patients giving a female:male ratio of 2.2:1. The mean age at DOT was 76.4 (SD=7.8) years of age (range 44–97 and median 76 years of age). The vast majority of patients (46.5%) were diagnosed in the seventh decade of life, with the eighth decade being the next most common age category in which patients present (30.6%) (online supplementary table 1 and supplementary figure 2). There was no statistical difference in the age at diagnosis between men and women (76.1 and 76.6 years of age, respectively).

10.1136/rmdopen-2017-000531.supp3 Supplementary file 3

10.1136/rmdopen-2017-000531.supp4 Supplementary file 4

In the Poisson regression, we modelled the number of GCA cases as a function of age, gender and season (table 2). Age and gender were both noted to influence GCA rate, with the number of events being more common in women and the elderly. Compared with women (reference category), men were found to have a 55% reduction (1–0.45) in the rate of GCA events (p<0.001). For age, compared with the youngest group of participants (40–59 years—reference category), there was a general trend towards increasing rates of GCA in older age groups. For example, those aged between 70 and 79 years were observed to have an 18-fold increase in the rate of GCA events compared with the reference category. There was no age–gender interaction on the rate of GCA events.

**Table 2:** Standard Poisson regression for all data (both hemispheres)

	Rate ratio	95% CI (lower)	95% CI (upper)	p Value
(Intercept)	10.17	7.66	13.22	<0.001
Season (ref=summer)				
Autumn	0.92	0.82	1.04	0.179
Spring	0.96	0.85	1.08	0.478
Winter	0.93	0.82	1.04	0.200
Gender (ref=female)				
Male	0.45	0.41	0.49	<0.001
Age (ref=40–59)				
60–69	6.93	5.28	9.26	<0.001
70–79	18.45	14.24	24.41	<0.001
80+	13.34	10.27	17.70	<0.001

The number of giant cell arteritis cases is modelled as a function of age, gender and season.

Ref, reference category.

**Figure 1:**
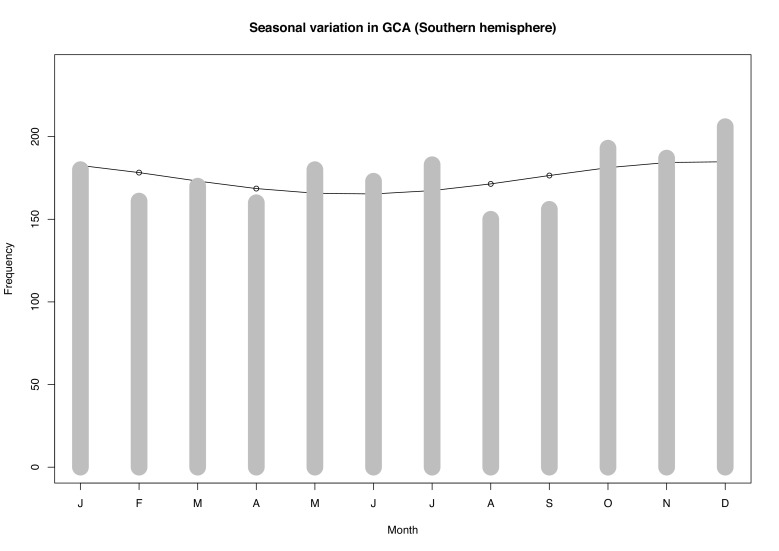
Cosinor test for Southern Hemisphere data (adjusted for age and sex). GCA, giant cell arteritis.

The distribution of TAB-positive cases across the four seasons was equal with approximately 530–580 cases being diagnosed each quarter (online supplementary file 5). Our data revealed no over-riding season in which the incidence of GCA predominated in Australia or New Zealand, and although our sample cohort from Northern Europe was small, there was no season in which TAB procedures prevailed more. Poisson regression analysis confirmed that there was no statistically significant evidence for an association between GCA rate and seasonal variation, after adjusting for age and gender. With summer as the reference category, the rate ratio of GCA approximated the null value (one) for each of autumn, spring and winter (table 2). Grouping the colder months (autumn and winter) versus the warmer months (summer and spring) in the Poison regression analysis did not significantly affect the rate ratio (0.94, p=0.17 with summer/spring as the reference category). Similarly, the results of the cosinor analysis reflected that of the Poisson regression (figure 1). While the amplitude of the cosine curve was noted to peak in the warmer months and trough over winter, this seasonal difference in GCA rate was marginal and not statistically significant. As such, both types of statistical models support the conclusion that we find no evidence for seasonal variation in the rate of GCA, after adjusting for age and sex.

10.1136/rmdopen-2017-000531.supp5 Supplementary file 5

### Systematic review

The results of our systematic review are summarised in table 3. Our search determined 104 publications. Following application of the inclusion criteria, we included 26 relevant articles investigating a wide range of environmental risk factors and their link with the development of GCA. Fifteen studies investigated the impact of seasons and/or annual cyclic trends and their influence on the onset of GCA, the results of which seem to be inconsistent. Other studies investigated latitude, altitude, solar exposure, socioeconomic status (SES) and urban versus rural living again with little conclusion.

**Table 3:** Summary of studies reviewing environmental factors in the onset of GCA

Environmental RF	Country	Years	Cases	Diagnosis	Outcome summary	Significant	Author
Seasons and annual cyclic trends							
Seasons	Australia	1992–2011	314	TAB	Summer (January/December) month increased incidence (p=0.015)	Yes	Dunstan *et al* 37
Seasons and infection	Denmark	1982–1994	–	TAB and clinical	Quarterly and annual fluctuations seen (association with infectious epidemics)	Yes	Elling *et al* 10
Seasons	France	1983–1995	109	TAB and clinical	No seasonal variation but peak in December	No	Ramassamy *et al* 40
Seasons	France	1993	213	–	No seasonal pattern	No	Raynauld *et al* 41
Seasons	Israel	1980–2004	210	TAB and ACR	Incidence more common late spring/early summer (p<0.001)	Yes	Bas-Lando *et al* 9
Seasons	Israel	1980–1991	84	TAB	Incidence more common during May to June (p<0.0005)	Yes	Sonnenblick *et al* 20
Seasons	NZ	1996–2005	70	TAB	Incidence greatest in spring (p<0.9)	No	Abdul-Rahman *et al* 8
Seasons	Spain	1981–2005	255	TAB	No seasonal pattern over 25 years	No	Gonzalez-Gay *et al* 33
Seasons	Spain	1976–2001	184	TAB and clinical	No cyclic or seasonal pattern of variation	No	Liozon *et al* 39
Seasons and cycles	Spain	1981–1999	161	TAB	No seasonal or annual incidence pattern seen in the Lugo region	No	Gonzalez-Gay *et al* 42
Season	Spain	1985–1997	143	–	No seasonal variation	No	Narváez *et al* 43
Seasons	Sweden	1997–2010	840	TAB	No seasonal or significant monthly variation	No	Mohammad *et al* 44
Seasons and cycles	Sweden	1976–1995	665	TAB	Random annual trends. Season predominance in winter and autumn (p=0.041)	Yes	Petursdottir *et al* 18
Seasons and cycles	USA	1994–2011	215	TAB	No significant fluctuations in the annual or monthly data (p=0.55).	No	Kisza *et al* 45
Seasons and cycles	USA	1950–1999	173	TAB and ACR	Cyclic incidence peaks 7–10 yearly, last ~3 years. No significant seasonal pattern	No	Salvarani *et al* 46
Latitude							
Latitude	Norway	1992–1996	70	TAB and ACR	No incidence difference between Northern and Southern Norway	No	Haugeberg *et al* 21
Latitude (and seasons)	UK	1990–2001	3928	Clinical (GPRD)	Incidence in South-North (UK), and greater in summer months (p=0.0022)	Yes	Smeeth *et al* 13
Altitude							
Altitude	Spain	1981–2001	–	TAB	Altitude had no effect on incidence of GCA (altitude ranged from 100 to 952 m)	No	Llorca *et al* 23
Solar cycle and sunlight							
Solar and geomagnetic	USA	1950–2004	–	–	Geomagnetic activity may explain temporal variation and east-west skewness	Yes	Wing *et al* 26
Solar exposure	Australia	1997	2	Skin biopsy	Solar aetiology: link between actinic granulomas and GCA of subcutaneous vessels	–	Lau *et al* 47
Solar exposure	Australia	1987	4*	Clinical and TAB	Observations: actinic radiation=vascular risk factor (elastotic/lytic properties)	–	O’Brien^48^
Solar exposure	UK	1965	60	Clinical	Sun exposure may cause GCA (14/18 cases sun exposed). Incidence up in June	–	Kinmont and McCallum^19^
SES							
Occupation and SES	Sweden	1964–2008	8019	Hospital register	Education, marital status, SES, occupation only weakly associated with GCA	No	Zöller *et al* 22
SES	UK	2005–2009	271	TAB and ACR	Area-level socioeconomic deprivation associated with ischaemia from GCA.	Yes	Mackie *et al* 29
SES, sunlight, infection	France	1970–1979	94	–	Environmental factors (sun exposure, lifestyle, SES) do not affect incidence	No	Barrier *et al* 49
Urban versus rural							
Urban versus rural	Germany	1994	79	ACR	GCA was more common in the urban than in the rural populations	Yes	Reinhold-Keller *et al* 30

Symbol (–) denotes unable to complete this information.

*Observational study with numbers not well defined.

ACR, American College of Rheumatology; GCA, giant cell arteritis; GPRD, General Practice Research Database; NZ, New Zealand; RF, risk factors; SES, socioeconomic status; TAB, temporal artery biopsy.

#### Seasons and annual cycles

Most of the non-infectious environmental studies in GCA cohorts have investigated the aspects of seasonality and/or cyclic fluctuations of GCA incidence. As demonstrated by our literature search findings (table 3), this has been a controversial theory; just one third (5 out of 15 studies have found a significant association between the onset of GCA to occur either within a specific season or that certain annual fluctuations are seen. The other studies demonstrated no significant seasonal pattern or cyclic trend to explain GCA incidence. For those studies that did find a peak within certain seasons or months, the trend was not consistent; some found more cases in summer while others in winter. A Swedish study described a peak in GCA incidence rate in autumn and winter,^18^ while studies in the UK and Israel describe a peak in spring and summer.^9 19 20^ There seems to be no overall consensus on seasonality and incidence rate of this disease.

#### Latitude and altitude

The increased risk to develop GCA in the Caucasian population was previously attributed to a possible theory on latitude, where moving away from the equator to more northerly latitudes might increase disease risk. A Norwegian study did not support this notion; regional differences could not be accounted for by a North-South gradient.^21^ In a Swedish study, a lower risk of GCA was found in Northern Sweden, suggesting that the high risk in Sweden is not caused by a colder climate alone.^22^ The potential role of altitude as a geographical factor has also been implicated in the incidence of GCA. Nevertheless, a Spanish study assessing altitude at the site of residence of 210 patients with GCA within the Lugo region revealed no difference in disease incidence related to this variable, nor did it account for relapse rates.^23^

#### Solar cycle and geomagnetic effects

The theory of sunlight as a risk factor for GCA has long been promulgated. In 1965, Kinmont and McCallum described 14 patients with GCA who suffered serious vascular complications after 'excessive' or 'merely unusual' sun exposure.^19^ They noticed that incidence was greatest in summer months and postulated that light sensitisation of the ageing skin to sunlight may provoke the acute phase of GCA.^19^ In 1978, O'Brien published evidence showing that long-term sun damage is common in the temporal arteries. He proposed that solar radiation destroys the essential supportive elastic tissue framework of arteries. As the temporal arteries run along the forehead they were vulnerable.^24 25^ In a recent US study, the impact of geomagnetic effects and solar cycle on the incidence of GCA in Olmsted County in Minnesota was investigated.^26^ They found that higher geomagnetic activity was associated with higher rate of GCA incidence. GCA rates peaked 0–1 year after strong magnetic activity, possibly suggesting that the effect is cumulative or that latency between environmental exposure and disease manifestation could be due to a complex autoimmune process.^26^ However, in this same study, the correlation between solar extreme ultraviolet radiation and GCA incidence was also investigated, and found that the correlation was not as significant as geomagnetic impact.^26^

#### Socioeconomic status

In a national Swedish study, educational level, family income, marital status and occupation seemed to have only a weak relationship with GCA.^22^ Interestingly though, in this study, certain comorbidities, such as hypertension and type 2 diabetes, placed people at higher risk of developing GCA.^22^ Other case–control studies have reported an increased risk of GCA in heavy smokers and in patients with previous atherosclerotic disease,^27 28^ but this was not replicated in the Swedish cohort.^22^ A British study found that area-level socioeconomic deprivation was associated with ischaemic manifestations resulting from GCA. This is most likely because individuals living in more deprived areas do not seek medical attention as early and hence are delayed at getting treatment.^29^

#### Urban versus rural living

Some studies have found a trend, although non-significant, that urban lifestyle may possibly predispose individuals at developing GCA.^22^ In a German study, in both Northern and Southern Germany, GCA has been significantly more prevalent in urban populations compared with rural populations.^30^ It remains unclear whether this disparity is due to underdiagnosis of GCA in the rural regions associated with differences in healthcare systems in cities versus rural areas.

## Discussion

To our knowledge, our GCA data set is one of the largest GCA data sets worldwide. Unlike most large epidemiological data sets available on GCA, our data were not obtained through retrospective search of primary care registers or other national available public health system databases.^4 31 32^ Instead, we analysed data gathered from the time of patients’ biopsies.

Our patient demographic is similar to previous studies. The female-to-male ratio was similar in both our Southern and Northern Hemisphere data sets (2.23 and 2.38 respectively). This is comparable to findings from a ‘meta-analysis’ comprising British, North American and Swedish populations stating that 70.2% of patients with GCA in the combined studies were female (ratio 2.39).^4^ In most studies, the female-to-male ratio has been reported at around 2.5. However, interestingly female predominance is less pronounced in Israel, Turkey and Mediterranean countries.^9 33–36^ The mean age of diagnosis in our study was 76.44 years. This is representative of data from other studies, indeed making it one of the latest onset systemic autoimmune diseases.^4^

Over the years, there have been numerous hypotheses regarding putative environmental risk factors for GCA, yet none have been robustly confirmed. Our study supports the view that there is no obvious seasonal trend of GCA in Australia and New Zealand. This is different from a recent study in South Australia which showed statistically significant higher rates of GCA observed in the Southern Hemisphere summer months, December to January,^37^ and a study in New Zealand which showed a trend for increased incidence in spring although not statistically significant.^8^ The theory on latitude also remains contentious. Latitude does not explain why Scotland has a lower GCA incidence rate than the rest of the UK,^38^ and why the incidence rates in Lugo (Spain) and Havsa (Turkey) are very different yet both locations are of very similar northerly latitude (43.0097°N and 41.5550°N respectively). As highlighted by the numerous studies in our systematic review (table 3), there seems to be no consensus on environmental risk factors and their impact on the incidence rate of this disease.

Our study has some limitations. Our cohort of GCA cases comprised TAB-positive cases only. As such, our data set does not include TAB-negative or clinically diagnosed GCA cases. In addition, we were dependent on the ability of pathology centres to easily access archived TABs as we required biological sampling for a concomitant genetics study. As such, those FFPE blocks stored offsite may have been unavailable or difficult to recruit and hence not collected. This may have led to a non-consecutive recruited data set. Although we are unable to comment on annual fluctuations and/or epidemic effects, given that the distribution of cases across the four seasons is very evenly spread with the TAB-positive cases only, the exclusion of the occasional clinically diagnosed GCA case and/or the exclusion of the irretrievable FFPE block would unlikely have altered our findings.

The season in which a patient was diagnosed was estimated based on the DOT. However, depending on how long a patient waits before seeking medical advice and/or is being referred for appropriate investigation, clinical diagnosis (onset of symptoms) may precede histological diagnosis by weeks, even months. As such, an important caveat of our work is that DOT may not represent the actual onset of disease in each case. However, presuming that the length of time between onset of symptoms and ischaemic manifestations from GCA is relatively uniform, as well as the fact that the number of cases across all seasons was evenly spread, it is unlikely that using DOT as a tool to categorise patients to seasons would have dramatically influenced our results.

Our data set was derived from the active participation of a selective group of hospitals and pathology centres rather than through a population-based study approach with access to a national database. Case recruitment was not necessarily representative of disease activity across Australia and New Zealand. Certain regions may have been over or under-represented. We lack samples and data predominantly from the tropical regions due to lack of expressed interest from potential collaborators in these areas. Of the 79 FFPE samples recruited from a pathology centre in Queensland, 69 were recruited from the Brisbane area in subtropical South East Queensland and only 10 from tropical North Queensland.

Because of this selection bias of participating pathology centres, we were unable to extrapolate the incidence rates of GCA from certain hospitals and/or pathology sites to accurately represent entire regions or states. As such, we were unable to comment on the intra or interstate variation in incidence rate of GCA; we were unable to analyse incidence based on latitude, nor compare incidence between wet and dry seasons in the more tropical areas.

It is also important to note that we only have data available on seasonal influence from two countries in Northern Europe. We incorporated their data and compared the influence of seasons on GCA onset between Australasia and Northern Europe. Although we accounted for seasons in the Northern Hemisphere being the reverse of those in the Southern Hemisphere, the climate of each season in the Northern Europe is likely different from that of its corresponding season in Australia and New Zealand.

Most of our demographic data were retrieved directly from pathology databases. Detailed clinical information from medical records, including details such as smoking status, ethnic background and address, was not retrieved. Some patients would have travelled from rural areas to have their TAB performed in tertiary referral centres. However, this information was not available and so we cannot determine whether our recruited population has an urban versus rural predisposition, and whether there is SES association.

Irrespective of some of the limitations we faced in the recruitment of our demographic data, studying the role of environmental risk factors in disease aetiology always proves challenging in view of numerous confounding factors, which are difficult to eliminate entirely. It is difficult to draw evidence on the true effect of latitude on the incidence of GCA as other factors come into play and are hard to dissociate.^39^ Even when comparing two populations of similar ethnic background living at two different latitudes within the same country, for example, Cairns and Hobart in Australia (16.9186°S and 41.4545°S respectively), factors such as timing of migration to Australia (ie, number of Generations Australian), mobility within Australia, lifestyle in these areas (eg, outdoor vs indoor activity), sun exposure, agricultural versus industrial workplace, fauna and flora may all influence risk.

In summary, this demographic GCA data set is one of the largest worldwide and certainly the largest in Australasia. Our results demonstrate equal distribution of the incidence rate across all four seasons and hence do not support a seasonal component contributing to the onset of disease. Alongside our geoepidemiological study, our systematic literature review highlights that there is no convincing and certainly no consistent evidence for role of direct environmental factors on the risk of developing GCA.

10.1136/rmdopen-2017-000531.supp6 Supplementary file 6
