# Supplementary material for: Geo-epidemiology of temporal artery biopsy-positive giant cell arteritis in Australia and New Zealand: is there a seasonal influence?
Source: RMD Open. 2017 Aug 29;3(2):e000531. doi: 10.1136/rmdopen-2017-000531 (PMC5706482; doi:10.1136/rmdopen-2017-000531)
Supplement: Supplementary file 1 [file rmdopen-2017-000531supp001.docx]

**Appendix B**. Ethic committees and approvals

The St Vincent's Hospital Melbourne Human Research Ethics Committee - HREC A 026/09; The Royal Victorian Eye and Ear Hospital Human Research Ethics Committee - 09/870H; The Melbourne Health Human Research Ethics Committee - 2014.012; Eastern Health Human Research Ethics Committee - E31/1314; The Monash Health Human Research Ethics Committee - 14426B; Alfred Health Ethics Committee - 96/14; The Austin Human Research Ethics Committee - LNR/14/Austin/151; The Royal Perth Hospital Human Research Ethics Committee - REG 14-003; The University of Western Australia Human Research Ethics Office - RA/4/1/5254; St John of God Health Care Human Research Ethics Committee - 716; Southern Adelaide Clinical Human Research Ethics Committee - 286.14 HREC/14/SAC/298; South Eastern Sydney Local Health District Human Research Ethics Committee 13/372 LNR/14/POWH/81; ACT health Human Research Ethics Committee - ETH.2.14.024; Royal Brisbane & Women's Hospital Human Research Ethics Committee - HREC/14/QRBW/429; University of Tasmania Human Research Ethics Committee - H0013881; the University of Otago Human Health Ethics Committee - 13/108.
