# Supplementary figures and images for: Geo-epidemiology of temporal artery biopsy-positive giant cell arteritis in Australia and New Zealand: is there a seasonal influence?
Source: RMD Open. 2017 Aug 29;3(2):e000531. doi: 10.1136/rmdopen-2017-000531 (PMC5706482; doi:10.1136/rmdopen-2017-000531)

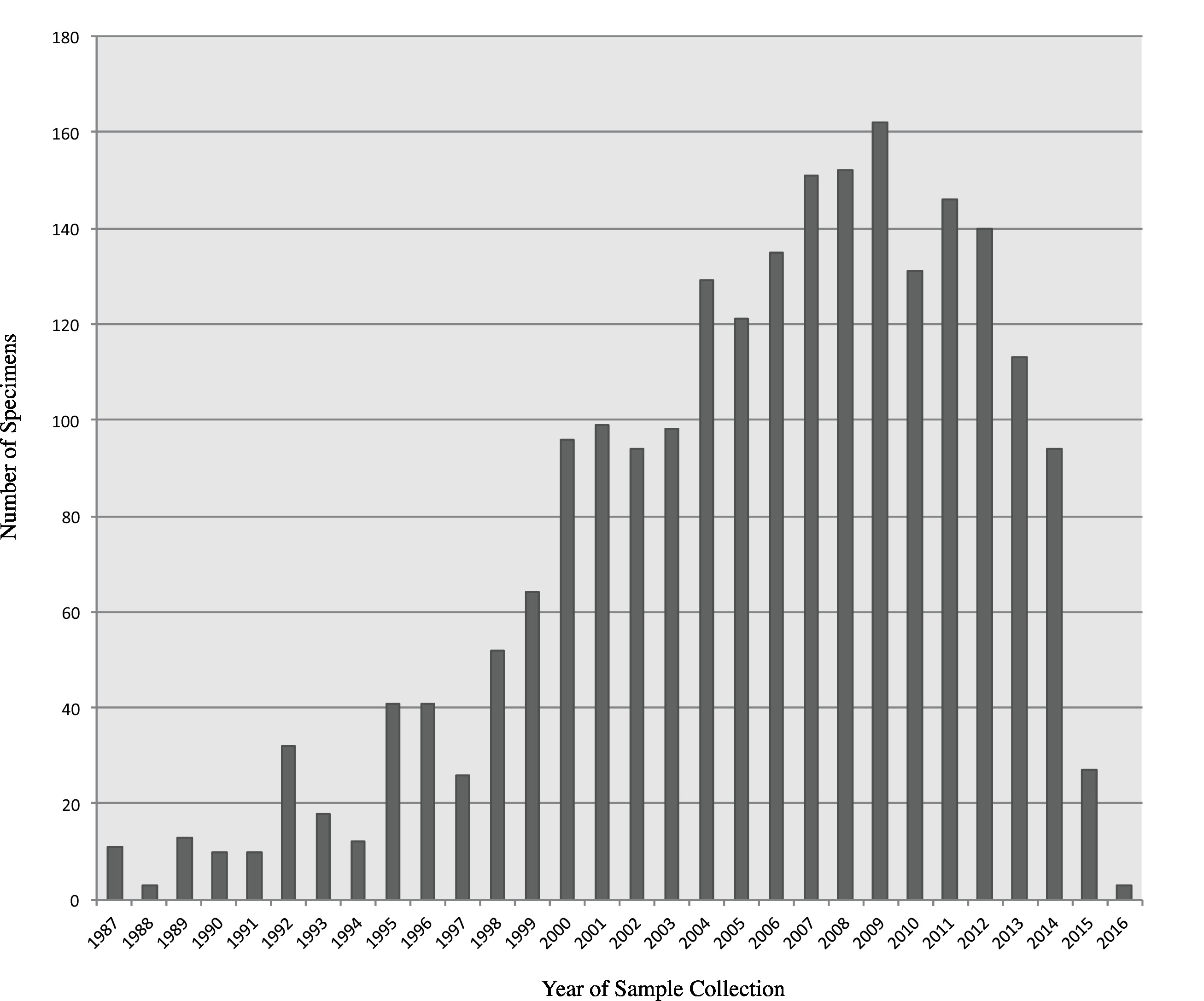

Supplement: Supplementary file 2 [file rmdopen-2017-000531supp002.jpg]

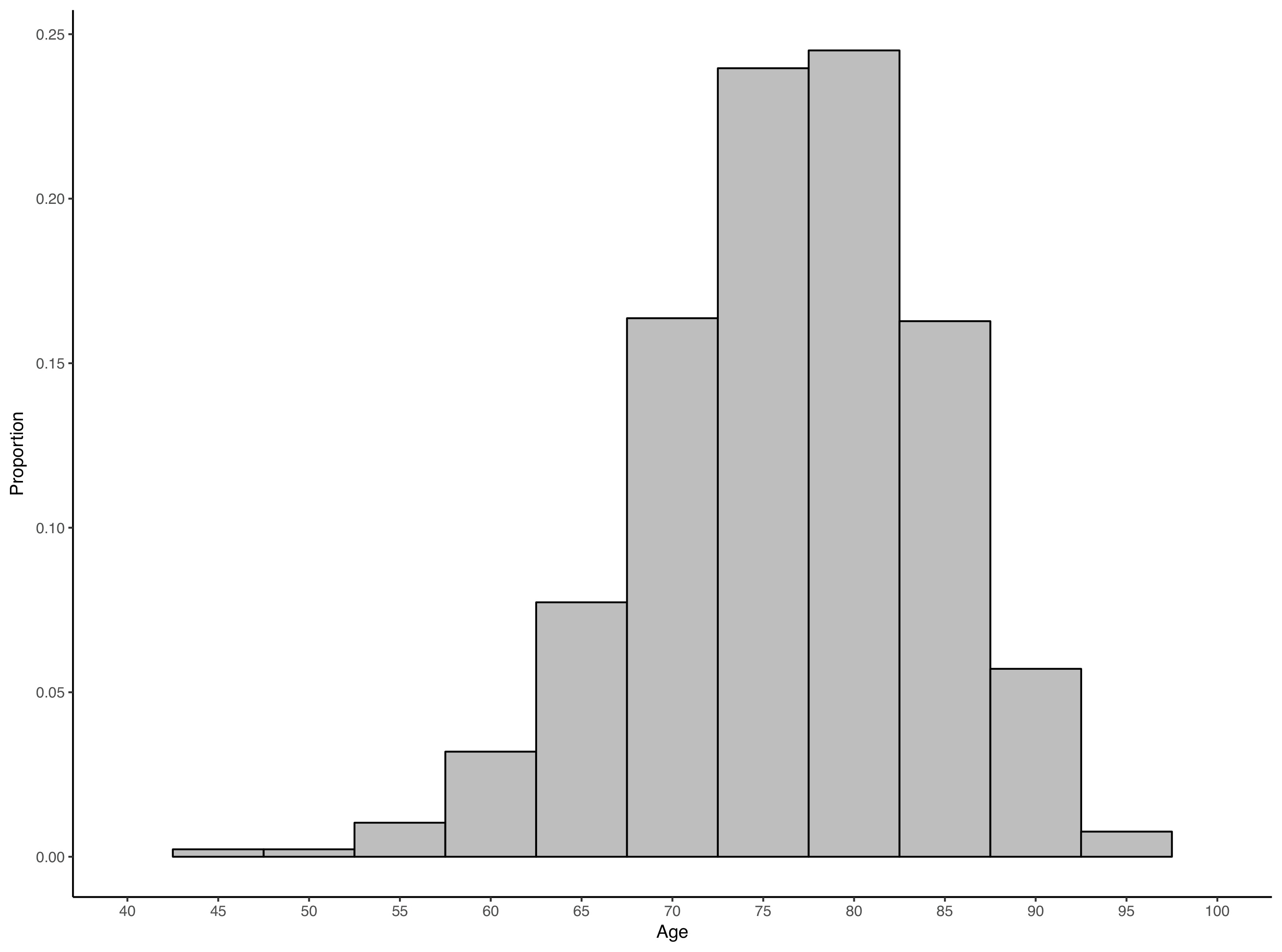

Supplement: Supplementary file 4 [file rmdopen-2017-000531supp004.jpg]
