# Supplementary material for: Geo-epidemiology of temporal artery biopsy-positive giant cell arteritis in Australia and New Zealand: is there a seasonal influence?
Source: RMD Open. 2017 Aug 29;3(2):e000531. doi: 10.1136/rmdopen-2017-000531 (PMC5706482; doi:10.1136/rmdopen-2017-000531)
Supplement: Supplementary file 3 [file rmdopen-2017-000531supp003.docx]

**Supplementary Table 1.** Number of patients recruited per age category

| **Age in years at time of disease onset** | **Southern Hemisphere Numbers (%)** | **Northern Hemisphere Numbers (%)** | **Total Study Numbers (%)** |
| --- | --- | --- | --- |
| <50 | 8 (0.4) | 0 (0) | 8 (0.36) |
| 50s | 48 (2.3) | 0 (0) | 48 (2.16) |
| 60s | 362 (17.2) | 26 (20.8) | 388 (17.45) |
| 70s | 971 (46.3) | 62 (49.6) | 1033 (46.45) |
| 80s | 646 (30.8) | 34 (27.2) | 680 (30.57) |
| 90s | 64 (3.0) | 3 (2.4) | 67 (3.01) |
| 100s | 0 (0) | 0 (0) | 0 (0) |
