# Supplementary material for: Geo-epidemiology of temporal artery biopsy-positive giant cell arteritis in Australia and New Zealand: is there a seasonal influence?
Source: RMD Open. 2017 Aug 29;3(2):e000531. doi: 10.1136/rmdopen-2017-000531 (PMC5706482; doi:10.1136/rmdopen-2017-000531)
Supplement: Supplementary file 5 [file rmdopen-2017-000531supp005.docx]

**Supplementary Table 2.** Season in which GCA cases were diagnosed,

|  | Southern Hemisphere | Northern Hemisphere | Both Hemispheres |
| --- | --- | --- | --- |
| Spring | 536 | 24 | 560 |
| Summer | 547 | 37 | 584 |
| Autumn | 510 | 29 | 539 |
| Winter | 506 | 35 | 541 |
