# Supplementary material for: Geo-epidemiology of temporal artery biopsy-positive giant cell arteritis in Australia and New Zealand: is there a seasonal influence?
Source: RMD Open. 2017 Aug 29;3(2):e000531. doi: 10.1136/rmdopen-2017-000531 (PMC5706482; doi:10.1136/rmdopen-2017-000531)
Supplement: Supplementary file 6 [file rmdopen-2017-000531supp006.docx]

**Appendix A.** Giant Cell Arteritis Pathology Network *

Benhur Amanuel, Michael Awadalla, Matt A Brown, Celia Chen, Svetlana Cherepanoff, Linda Clarke, Jamie Craig, Katie Cremin, Erika de Guzman, Elisabeth De Smit, Jonathan Dick, Mitali Fadia, Cindy Forrest, Anthony Gill, Ruta Gupta, Helen Harris, Andrew Harrison, Alex W Hewitt, Catherine L Hill, Claire Hooper, Patrick Hosking, Jost Jonas, Lisa S Kearns, Sonja Klebe, Anthony Landgren, Soon Lee, Trische Y-M Leong, David Mackey, Roslyn Malley, Peter McCluskey, Daniel Mckay, Penny McKelvie, Catriona McLean, Tony Merriman, Andrew Miller, Andrulis Mindaugas, Logan Mitchell, Vince Murdolo, Min Ru Qiu, Thomas Robertson, Meena Shingde, Lisa Stamp, LayKhoon Too, Rob Verdijk, Tersia Vermeulen, Owen White, David Williams, Christine Younan, Nikolajs Zeps
